# Do We Need a New Approach to Cancer Biology Education for Radiation Oncology Residents?

**DOI:** 10.7759/cureus.20662

**Published:** 2021-12-24

**Authors:** Srinivasan Vijayakumar, Maurice King, Mary R Nittala, Roy J Duhe

**Affiliations:** 1 Radiation Oncology, University of Mississippi Medical Center, Jackson, USA; 2 Pharmacology, University of Mississippi Medical Center, Jackson, USA

**Keywords:** radiobiology, cancer biology education, future radiation oncologists, radiation & medical oncology, precision medicine

## Abstract

Traditional radiation oncology biology courses largely focus on radiation biology and oncology as needed for passing the boards. Changes in the landscape of oncology necessitate a broader scope. Radiotherapy is an important component of cancer care. Approximately 70% of all cancer patients receive radiotherapy during the course of their disease. With the revolution in precision medicine that is unfolding, genomics, proteomics, metabolomics, and microbiomics are being ever more integrated into the treatment of cancer. Comprehensive knowledge of cancer biology beyond traditional radiation biology is essential for future advances in radiotherapy and unavoidable for radiation oncology trainees. The importance of a newly designed curriculum to impart broader knowledge to future radiation oncologists is emphasized in this report. A paradigm shift in the approach to the traditional radiation biology course is required to train residents for the future of oncology.

## Editorial

Background

The impact of cancer on all our lives emphasizes the need for continuous training to pursue research into its cure and prevention. Radiation oncology has an excessive degree of benefit to cancer patients, but it is very important to understand the effect of radiation as a potent modulator of the genetic and cellular activity of cancer. The main objective of this commentary is to describe the requirement in having a profound impact on how cancer biology education is valued for radiation oncology residents and by providing cancer-focused educators with the ability to develop a robust cancer education and comprehensive career development programs.

Discussion

Oncology has entered a new era. Technical advances in collecting and analyzing data in the fields of genomics, proteomics, metabolomics, and microbiomics are fundamentally redefining our understanding of cancer and how to treat it. Massive amounts of genetic data procured through such initiatives as the Cancer Genome Atlas [1], the 100,000 Genomes Project [2], and the American Association for Cancer Research (AACR) Project Genomics, Evidence, Neoplasia, Information, Exchange (GENIE) [3], have become increasingly annotated with clinical data. These Herculean efforts have resulted in a quantum leap in our understanding of the genetic drivers of diverse cancers [4] and illuminated a myriad of fundamental cancer biology concepts [5-7] right down to the individual level. This has led to the development of new targeted agents, and patients now regularly undergo genetic sequencing to help guide their treatment [8]. We are in the age of precision medicine. A detailed and comprehensive understanding of cancer biology will become essential to the practice and future advancement in all fields of oncology.

Gone are the days when faculty could focus on radiobiology only for basic science didactic purposes. We must move beyond focusing solely on radiation’s effects on a cell and start considering the impact that an individual cancer’s cell biology and its tumor microenvironment will have on radiation therapy. Most residents enter radiation oncology programs with limited knowledge about cancer biology and may be somewhat unprepared to reap the benefits of the world of big data, which emanated from the Human Genome Project [9].

If “genomically guided radiation therapy is a necessity that must be embraced in the coming years” [10], we must prepare now. How can faculty provide an essential educational foundation without overwhelming radiation oncology residents? How can our residency programs better prepare our trainees for a future in which information technology constantly pushes the frontiers of precision medicine [11]? How can we arm our residents with the ability to discriminate between experimental radiation and pharmacological combination therapies which are based on sound scientific principles from those based on wishful thinking?

We recently attempted to address these and related concerns through a new Cancer Biology for Radiation Oncologists course. The course structure is designed to embed a lasting framework of fundamental principles in the minds of budding radiation oncologists so that they may be better able to apply guaranteed future advancements in technology, biology, and pharmacology to their medical practices. The course also addresses topics of immediate relevance to radiation oncologists, such as DNA repair mechanisms and molecular pathways providing radiation resistance.

Many uncertainties contribute to anxiety about the future of radiation oncology, and the specter of taking the American Board of Radiology Initial Certification Exam looms large in the minds of trainees at many institutions. Our new course devotes considerable attention to content which is unlikely to appear on this or other national exams, yet this course has been enthusiastically embraced by our residents.

**Figure 1:**
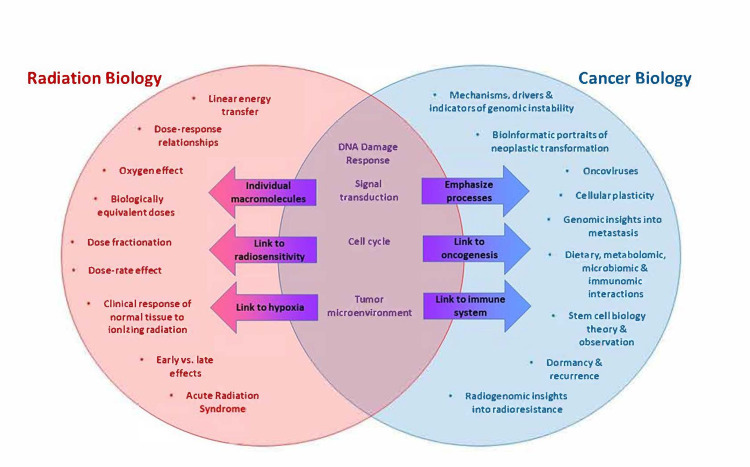
Comparison of themes emphasized by traditional radiation biology courses versus cancer biology courses tailored for future radiation oncologists

In Figure 1, traditional radiation biology courses (shown on the left in red) emphasize themes that allow trainees to fully understand the biomedical responses of tumors and patients to various doses of ionizing radiation under every conceivable dose delivery modality. Cancer biology courses tailored to future radiation oncologists (shown on the right in blue) emphasize themes that allow trainees to fully understand the various processes of neoplastic transformation and progression, the roles of critical genes and gene products in these processes, and their relationships to personalized medicine. Certain concepts (shown at the intersection in purple) are common to both courses, although their associated learning objectives may differ due to divergent relationships to other concepts taught in these courses (arrows).

Conclusions

We believe our enhanced approach to cancer biology education can help solve some of the challenges facing the radiation oncology community at large, but more importantly, improve health outcomes for the patients we all serve. It is important to note that cancer biology courses tailored to future radiation oncologists are not intended to replace radiation biology courses; rather, they are intended to supplement such courses.
